# Preparation, characterization and degradation study of novel sulfonated furanic poly(ester-amide)s

**DOI:** 10.1080/15685551.2020.1727171

**Published:** 2020-02-11

**Authors:** Majdi Abid, Sirine Mhiri, Abdelkader Bougarech, Rania Triki, Souhir Abid

**Affiliations:** a Chemistry Department, College of Science and Arts, Jouf University, Alqurayyat, Saudi Arabia; b Laboratoire De Chimie Appliquée H.C.G.P., Faculté Des Sciences De Sfax, Université De Sfax, Sfax, Tunisia

**Keywords:** Polyesteramide, polycondensation, bio-based polymers, hydrolytic degradation

## Abstract

A series of novel-sulfonated furanic polyesteramides have been synthesized by polycondensation in the melt-phase. The copolymers were obtained with reasonable molecular weights and high inherent viscosities between 0.2 and 0.4 dL/g. The hydrolytic degradability of polyesteramides was enhanced by the increasing of amide units in the copolymers backbone.

## 1. Introduction

Polymeric materials synthesized from renewable monomers have already gained an established position among synthetic materials both in academia and in industry. Predominantly due to two principal major reasons (i) firstly environmental concerns and (ii) secondly the realization that our petroleum resources are finite. In this context, several biosourced chemicals such as furanic, isosorbide, succinic acid and itaconic acid … monomers are considered as alternative, from the moment they make possible to obtain environmentally friendly polymers and with properties similar to their fossil counterparts [1–11]. In this part, we will focused on sulfonated bio-based polyesters and bio-based poly(ester-amide)s. Due to their intrinsic biological properties including biodegradability, biocompatibility and their basically easy synthesis, a particular interest has been given, in the last few years, to bio-based sulfonated polyesters [12–18]. Our team has been investigated the synthesis of new biobased sulfonated copolyesters from difuranic monomer with the dimethyl 5-sodiosulfoisophthalate and various diol units 1.2-ethanediol, 1.4-butanediol, 1.6-hexanediol, diethylene glycol or triethylene glycol. The resulting copolyesters exhibit a block distribution of furanic and sulfonic acid units and the final compositions attained in these materials were close to those of their feed. The thermal properties, sorption abilities and the hydrolytic degradation of these copolyesters were investigated and the study clearly showed the major role of the diol structure and the content of sulfonated units [12, 19]. Bautista et al. [20] report the synthesis of a series of sulfonated poly(butylenes succinate) (PBS) ionomers containing up to 14 mol% of sulfonated succinate units via polycondensation in the melt phase. All copolyesters were semi-crystalline with melting temperatures and enthalpies decreasing and glass transition temperatures increasing with the content of ionic units. The hydrolytic degradation of these ionomers was also performed in an aqueous medium at different pH to estimate their susceptibility to hydrolysis in different conditions. Recently, our team describe the synthesis and characterization, of fully biobased aliphatic sulfonated oligoesters based on diethylsuccinate (DES), 1.18-octadec-9-enedioic acid with **z** configuration(C18), hydrogenated dimer fatty acid (DA), sodium (sulfonated dimethyl succinate) (Na-DMSS), and two biosourced diols: 1.4-butane (BD) diol and isosorbide(Is). ^1^H-NMR spectroscopy and MALDI-TOF MS of the resulting oligoesters highlighting a regular structure. The thermal properties indicate that the ensuing oligoesters are amorphous or semicrystalline that essentially depend on the nature of monomers [21]. On the other hand, polymers with ester and amide groups in their skeleton new opportunities for incorporating biodegradable and adequate performance properties in a single material. In addition, the final characteristics can easily be adjusted by changing the amide/ester ratio or even the hydrophilicity of the polymer. Recently, particular attention was paid to samples derived from α-amino acids and renewable resources such as plant biomass and carbohydrates. A general assortment of the most original and interesting applications that have just been developed (for example, controlled drug delivery systems, non-viral delivery vectors, hydrogels, tissue engineering and intelligent materials) is also provided [22, 23].

Few studies concerning the preparation of biobased poly-esteramides have been carried out, for example, from saccharide by the polycondensation of bis(p-nitrophenyl) aliphatic dicarboxylates with p-toluene sulfonate salts of isosorbide or isomannide diesters of natural amino acids [24–26]. Recently, Triki et al. [27, 28] have found that the synthesis of furan polyesteramides (PEAF) by simultaneous high-temperature polyesterification and polyamidation of difurandiesters with hexane-1.6-diamine and ethane-1.2-diol conduct to novel structures of poly(ester amide)s PEAs. Using ^1^ H-NMR spectroscopy, they detected minor side reactions [24]. These same authors have studied the preparation of furanic-aliphatic polyesteramides from a furan diamine and adipic acid by two mass polycondensation techniques: the first one is based on the direct copolycondensation of monomers, namely the reaction of 5.5ʹ-Isopropylidene-bis(2-furfurylamine) (DAF) and ethanediol with diethyl adipate, and the second involves the aminolysis of an aliphatic polyester by DAF, followed by a polycondensation step [28, 29].

As a continuation of our studies on this family of polymer, a series of furanic poly(ester-amide)s bearing sulfonated groups in the main chain were synthesized from 5.5ʹ-Isopropylidene-bis(ethyl-2-furoate), dimethyl 5-sodiosulfoisophthalate, ethylene glycol and hexamethylene diamine by melt polycondensation using zinc acetate as a catalyst. These copolymers have potential interesting attributes: their biosourced origin, their relatively high thermal stability, the strong intermolecular hydrogen bonding interactions between amide functions and water molecules increases the hydrophilicity of the macromolecular chains and consequently their hydrolytic degradation. The hydrolytic degradation in acidic aqueous conditions (pH = 4.35) at 37°C over the period of 4 weeks show that the mechanism of the hydrolysis of poly(ester-amide)s was elucidated in relation with the microstructure.

## 2. Experimental

### 2.1. Materials

Dimethyl 5-sodiosulfoisophthalate (Na-DMSI) (98.00%), 2-ethyl furoate (99.00%), hexane-1.6- diamine (HMD, 99 + % Fluka), ethylene glycol (EG) (99.80%), tetrabutoxytitanium Ti(OBu)_4_ (97.00%), zinc acetate (Zn(OAc)_2_) (99.99%), and sodium acetate (NaOAc) were purchased from Sigma-Aldrich.

All chemicals were used as received, without further purification.

#### 2.1.1 Synthesis of 5.5ʹ-Isopropylidene-bis(ethyl 2-furoate) (DEBF)

The synthesis of 5.5ʹ-Isopropylidene-bis(ethyl 2-furoate) (DEBF) followed a previously reported procedure [7]. Typically, DEF was prepared by the condensation of ethyl 2-furoate (30 g) with acetone (9.39 ml) under acidic conditions (H_2_SO_4_, 30 ml), at 60°C for 8 h. The ensuing product was isolated in 95% yield by extraction, double distillation and recrystallisation from hexane.

## 3. Copolymerization

### 3.1. Synthesis of poly (ethylene bisfuroate-co-ethylene isophthalatesodiosulfonate) (PEBF-Co-PESI)

The polyesters were prepared by polycondensation in the melt from a mixture of variable amount of DEBF (4.68 mmol), Na-DMSI (1.17 mmol) with excess quantity of EG (17.5 mmol).

In the first step, the transesterification was carried out, under nitrogen atmosphere for 6 h at 200°C using Ti(OBu)_4_(0.1 wt%) as a catalyst. In the second step, the reaction was performed under reduced pressure for 6 h at 240°C. The resulting polymers are mentioned to as PEBF-Co-PESI.

### 3.2. Synthesis of polyamidesulfoisophatalate (PASI)

DEBF (4.7 mmol) and Na-DMSI (2 mmol) and excess of HMD (20.1 mmol) were charged to a 50-mL, threenecked, round-bottom flask equipped with a mechanical stirrer, a nitrogen inlet, and a distillation column. Zn(OAc)_2_(0.1 wt%) was added to the mixture to act as a transesterification catalyst. The reaction was carried out at 200°C under a nitrogen flow. Methanol and ethanol formed in the transesterification reaction was continuously removed by distillation for a period of 6 h. The excess of HMD was removed by high vacuum distillation at temperature ranged from 70°C to 200°C. The pressure was slowly reduced to 0.01 mbar and reaction continued for 4 h at 220°C. PASI was analyzed without further purification.

### 3.3. Synthesis of polyesteramide-sulfoisophatalate (PEASI)

Polyesteramides PEASI_1-3_ were prepared according to the general procedure described below for PEASI 1: DEBF (6.2 mmol), ED (35 mmol), Na-DMSI (2.6 mmol) and HMD (1.76 mmol) were introduced in a 50-mL glass kettle equipped with a central mechanical stirrer, a nitrogen inlet, a distillation head connected to a condenser and a receiver flask. The kettle was placed in a salt bath at 200°C. After 5 min of heating, the reaction medium was homogeneous and10^−3^ of total mass of zinc acetate Zn(OAc)_2_ was introduced in the mixture. Bath temperature was raised gradually to 220 C and ethanol distilled off. After 6 h heating, pressure was slowly reduced to 0.07 mbar and reaction continued for 4 h. PEASI_1_ was analyzed after cooling, without further purification.

## 4. Analytical techniques

^1^H, ^13^C NMR spectra were recorded in DMSO-d_6_ using a Bruker Avance III 400 spectrometer at 90°C. All chemical shifts (δ) downfield from the tetramethylsilane TMS (used as the internal standard).

Infrared spectra were recorded on a PerkinElmer 2000 Fourier transform infrared (FTIR) spectrophotometer at room temperature from powder pressed pellet samples in KBr. For each sample, 64 scans at a resolution of 1 cm^−1^ were collected and signal-averaged.

The inherent viscosities of the polymers were measured from DMSO solutions with an Ubbelohde viscometer maintained at 25.0 ± 0.1°C.

The hydrolytic degradation essays were performed with films prepared via solvent casting process.

After immersion in water (pH = 4.35) at 37°C for a fixed period of time, the samples were rinsed thoroughly in water and dried to constant weight. Sample weighting was used to follow the evolution of the hydrodegradation.

Films referenced as film PEASI_1-3_ and film PEBF-Co-PESI were prepared by casting a sample of PEASI_1-3_ polyesteramides and PEBF-Co-PESI polyesters at room temperature from a 10% (W/V) solution in CHCl_3_/MeOH (8/1 V/V) on silanized Petri dish. The films were cut and dried in vacuum at 50°C to constant weight. The thickness of the obtained films was 210 ± 10 mm.

## 5. Results and discussion

In view of the complexity of the ^1^H-NMR spectrum relating to the final PEASI copolymer, we first proceeded to prepare the homopolyester and the homopolyamide separately, assign the different signals on their spectra, and then the attribution of the signals of the PEASI spectrum.

### 5.1. Synthesis of PEBF-Co-PESI

The synthesis of poly(ethylene bisfuroate-co-ethylene isophthalate sodiosulfonate) PEBF-Co-PESI the two-step melt polycondensation method (transesterification and polycondensation)between 5.5ʹ-isopropylidene-bis(ethyl 2-furoate) (DEBF), dimethyl 5-sodiosulfoisophthalate (Na-DMSI) and 1.2-ethanediol (ED). The first step was carried out under a nitrogen atmosphere in the presence of zinc acetate as transesterification catalyst and the sodium acetate as inhibitor of etherification side reaction. The temperature was gradually raised from 180°C to 200°C during this step in order to avoid sublimation of reagent and side reactions. Until no more methanol and ethanol were collected, this step was considered to be over, typically within 3 h. The second step was completed at higher temperature (240°C) under vacuum in order to release the excess of diol and to shift the reaction equilibrium toward the formation of high molecular weight. This latter step, i.e. the polycondensation was catalysed by the addition of Ti(OBu)_4_. Finally, the reaction pressure was returned to normal atmospheric pressure by the introduction of N_2_ and the target product was obtained.

**Scheme 1.**
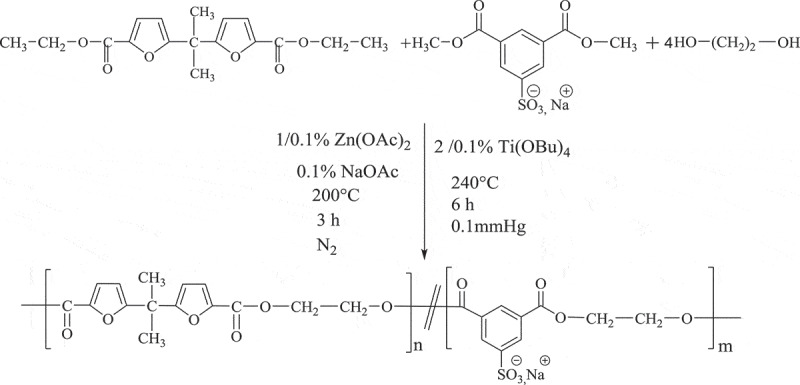
Synthesis of copolyester **PEBF-Co-PESI.**

**Figure 1.**
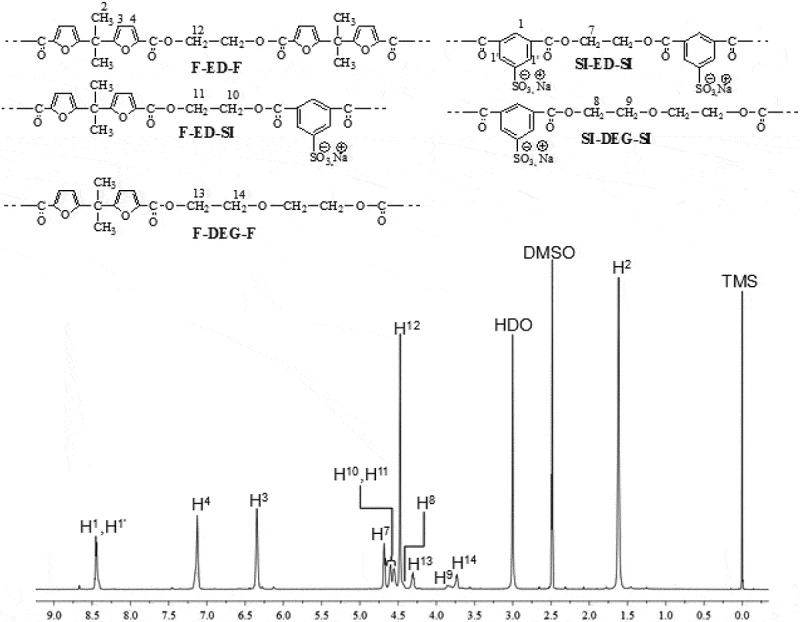
^1^H-NMR spectrum [400 MHz, DMSO-d6, reference: δ (TMS) = 0 ppm] of **PEBF-Co-PESI.**

The structural characterization of the PEBF-Co-PESI copolyester was performed by ^1^H-NMR. The molar fraction of furoate (*F*_F_) and sodiosulfoisophthalate (*F*_SI_) units in copolymers were determined from the integration of isophthalate (H^1,1ʹ^) and furoate (H^3,4^) NMR peaks (Figure 1). The presence of four resonances in 4.5–5 ppm region (assigned to the ethylene units of three different triads F-ED-F, F-ED-SI and SI-ED-SI) indicates that copolymerization took place. In addition to the expected ethylene glycol units, diethylene glycol (-CH_2_-CH_2_-O-CH_2_-CH_2_) units are present in the copolyester.

### 5.2. Synthesis of PASI

The sulfonated polyamide PASI was prepared as shown in by copolycondensation of 5.5ʹ-isopropylidene-bis(ethyl 2-furoate) (DEBF), dimethyl 5-sodiosulfoisophthalate (Na-DMSI) and an excess of hexane-1.6-diamine (HMD), in the presence of Zn(OAc)_2_ as catalyst. The reaction was first carried out under atmospheric pressure, then under vacuum with (HMD) elimination to obtain the final high-molar-mass of sulfonated polyamide.

**Scheme 2.**
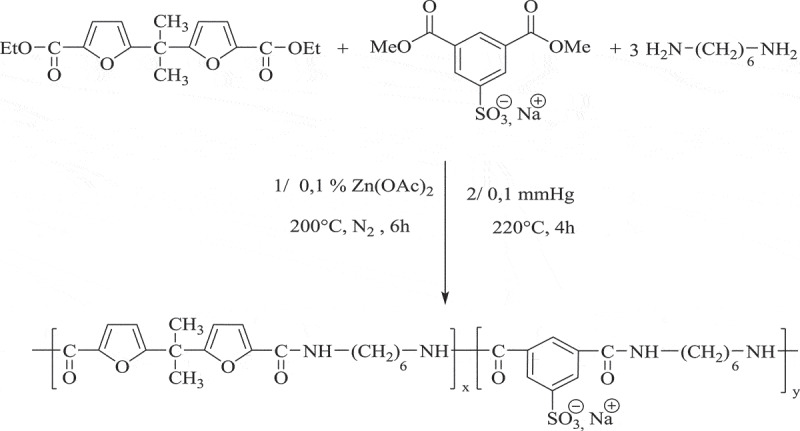
Synthesis of sulfonated polyamide **PASI.**

**Figure 2.**
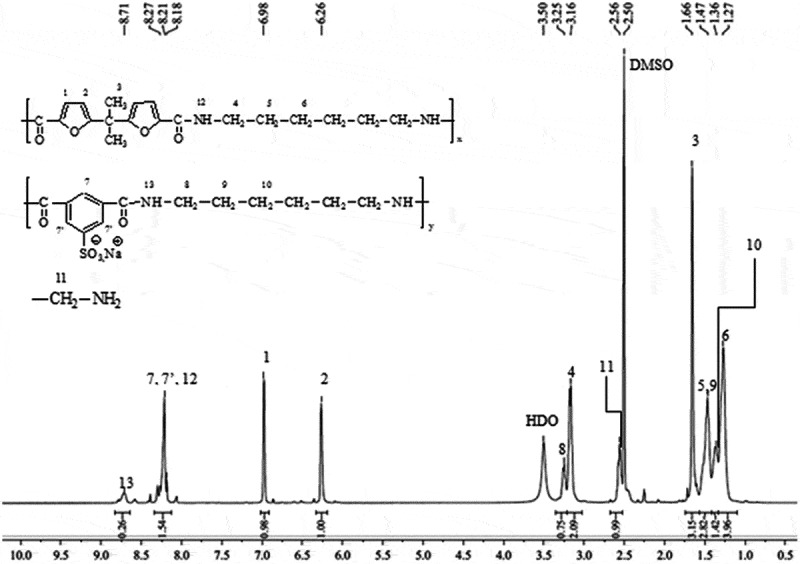
^1^H- NMR spectrum [400 MHz, DMSO-d6, reference: δ (TMS) = 0 ppm] of **PASI.**

For PASI, the ^1^H-NMR spectrum shown in Figure 2 highlights the expected signals of the furoate protons H^1^, H^2^ and H^3^ at 6.26, 6.98 and 1.66 ppm, respectively, and those of sodium sulfoisophthalate protons H^7^, H^7ʹ^ at 8.45 ppm. The (1.27–1.47) ppm and (3.16–3.25) ppm region of the ^1^ H-NMR spectrum, the resonances of amide methylene are easily assigned.

The amide proton (-NH) of sulfonated and furanic units (H^13,12^) are observed at 8.21 and 8.71 ppm, respectively.

### 5.3. Synthesis of sulfonated polyesteramide PEASI_1-3_

The sulfonated polyesteramides PEASIs were synthesized as shown in by simultaneous high-temperature polyesterification and polyamidation of difuranic diesters and dimethyl 5-sodiosulfoisophthalate with hexane-1.6-diamine and ethane-1.2-diol. The reaction was first carried out under atmospheric pressure in the presence of Zn(OAc)_2_ as catalyst, leading to oligoesteramide formation with ethanol and methanol elimination, then under vacuum with ED elimination to obtain the final high-molar-mass polyesteramide.

**Scheme 3.**
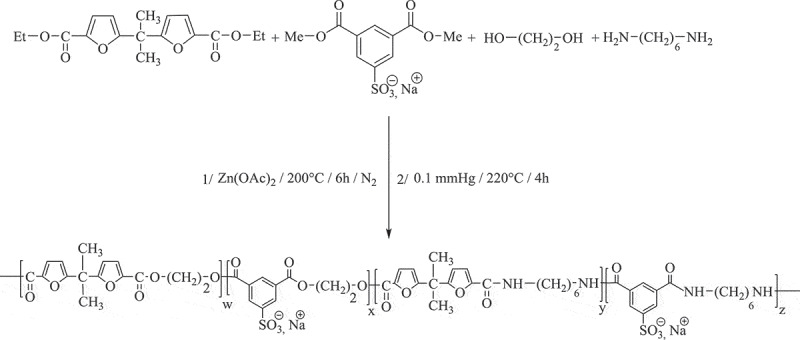
Synthesis of sulfonated polyester-amides **PEASI_1._**

**Figure 3.**
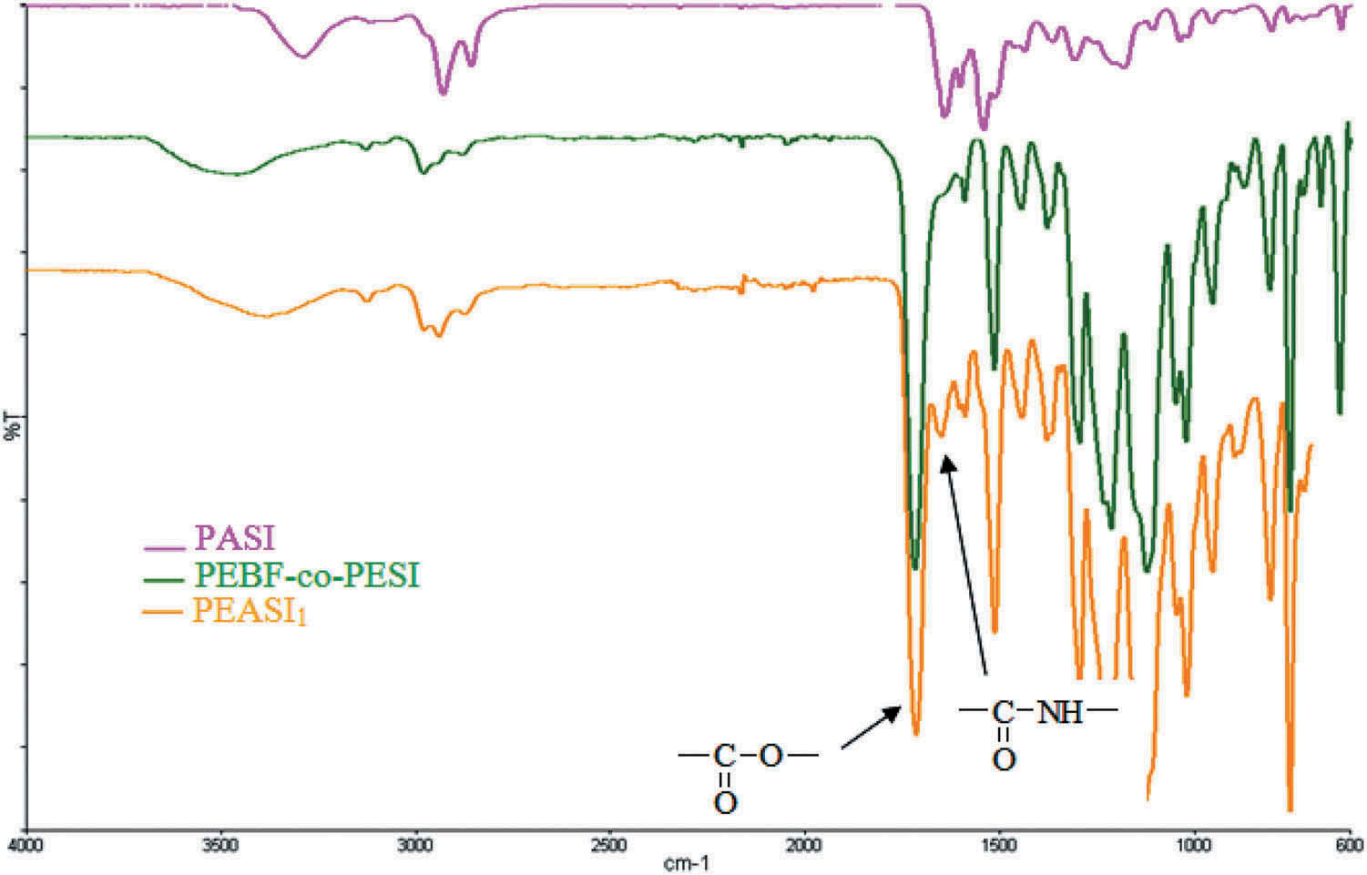
FT-IR spectrum of sulfonated polyester-amides **PEASI_1._**

We first concentrated on a specific monomer composition, DEBF/Na-DMSI/HMD/ED (0.7/0.3/0.2/4), leading to PEAS_1_, and then extended the study to other compositions to obtain a series of sulfonated PEASIs with various ester/amide ratios (Table 2). The evolution of the reaction is followed by spectroscopic analyses. The FTIR spectra, of PEASI_1_ show the characteristic bands absorptions of the carbonyl amide function at 1651 cm^−1^,while the carbonyl ester function 1715 cm^−1^ (Table 1). A representative FTIR spectrum of sulfonated polyester-amides PEASI_1_ is reproduced in Figure 3.

**Table 1.** FTIR spectral data of sulfonated polyester-amides **PEASI_1._**

	Functional groups (cm^−1^)											
Polymers	CH(Fu)	CH_2_	Amide I	Amide II	Amide III	C-O-C (Fu)	Respiration (Fu)	Fu 2,5 disub.	CH_3_	C-S	S-O	
PEASI_1_	3128	2874	1651	1513	1294	1126	1020	754 805 953	2980 2940	625	1212	1715

**Table 2.** Synthesis of polyesteramides **PEASI_1_** to **PEASI_3_**: initial DEBF:Na-DMSI:HMD:ED monomer composition mol ratio, ester:amide mol ratio in final PEAFs and inherent viscosity

Polymers	PEASI_1_	PEASI_2_	PEASI_3_
Initial DEBF:Na-DMSI:HMD:ED monomer composition mol ratio	0.7: 0.3:0.2:4	0.7: 0.3:0.5:4	0.7: 0.3:0.8:4
Final polymer composition Ester: Amide mol ratio	82/18	53/47	29/71
ŋ inh (25°C, DMSO) (dL/g)	0.2	0.35	0.4

**Figure 4.**
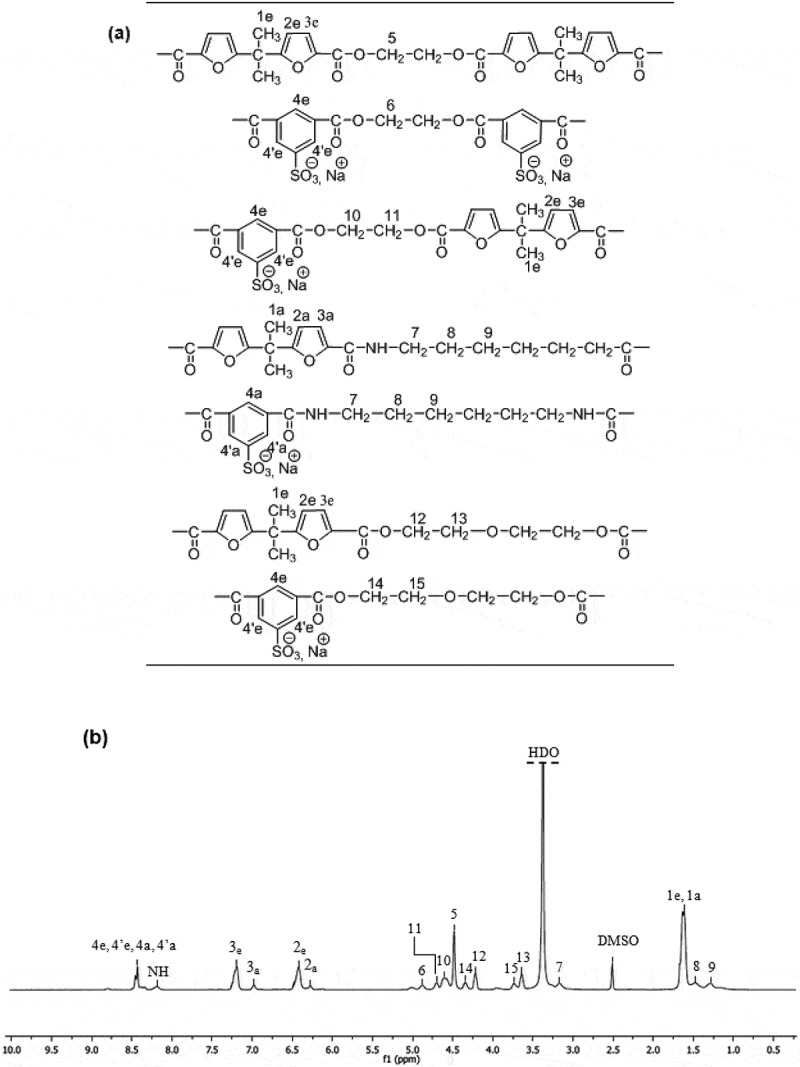
^1^H-NMR characterization of **PEASI_1_**,(a) atom numbering in sulfonated polyesteramides, (b) ^1^H NMR spectrum [400 MHz, DMSO-d6, reference: d (TMS) = 0 ppm]

The resonances of the ^1^H-NMR spectrum of PEASI_1_ were assigned by comparison with the spectra of pure polyester and pure polyamide (Figure 4). The resonances between 6–7.5 ppm region and 8.4 ppm correspond to the furanic and aromatic protons of amide and esters groups, respectively. In the aliphatic region, between 3.5 and 5 ppm the resonances of ester methylene are easily assigned. The resonance at 1.29, 1.48 and 3.17 correspond to the amide methylene. The appearance of signals corresponding to the amide alpha-methylene and – NH reflects the complete insertion of diamine in polyester chains. The relative integrations of resonances H^2e^, H^3e^, H^2a^ and H^3a^ lead to ester: amide mol ratio in final polymer very close to the theoretical one for all polyesteramides. The amide units have a significant effect on the increase of the inherent viscosity level from 0.2 to 0.4 (dL/g) arising the stiffening of the polymer backbone (Table 2).

### 5.4. Hydrolytic degradation

**Figure 5.**
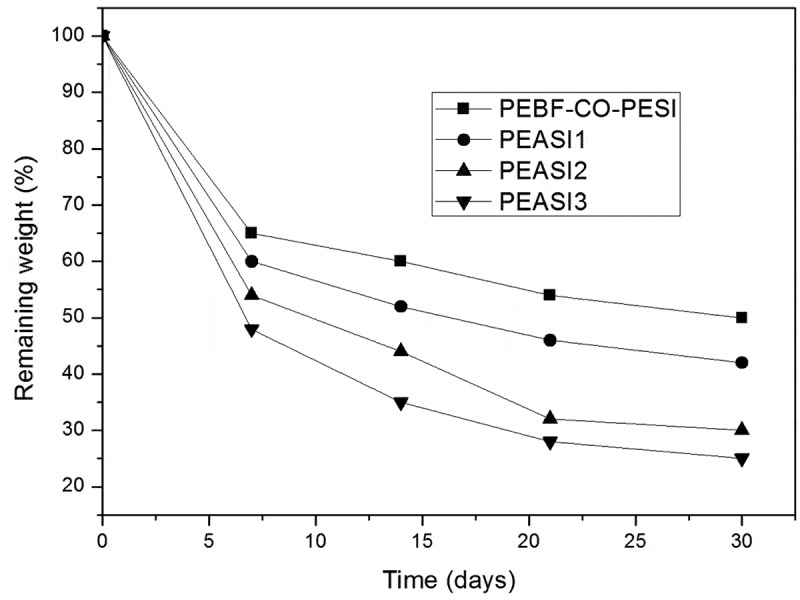
Hydrolytic degradation of **PEASI_1-3_** and **PEBF-Co-PESI**: Remaining weight (%) of samples immersed in water pH = 4.35 at 37°C

It is evident that polyester-amides can be influenced by chemical hydrolysis under acidic conditions. PEASI_1-3_ were submitted to hydrolytic degradation in acidic aqueous conditions (pH 4.35) at 37°C over a period of 4 weeks. After this period, each sample was rinsed thoroughly in water and dried to constant weight. The remaining weight of PEASI_1-3_ showed an overall decreasing trend over the entire period of degradation at (pH 4.35) (Figure 5).

As expected the remaining weight of the degraded samples of PEASI_1-3_ are minor than theirs homologous polyesters PEBF-Co-PESI. Obviously, the decrease of the remaining weight depend on the sulfonated unit but as can be seen in Figure 4 the polyester-amides having the highest amide molar ration led to the lowest value with a quasi plateau at around 25%. It was clearly shown that the introduction of amide functions promotes the hydrolytic degradation of the resulting copolymers.

## 6. Conclusion

We investigated the synthesis of sulfonated bio-based polyesteramides by step-growth polymerization in melt conditions. Experimental results indicated that the resulting polyesteramides exhibited a regular structure, high inherent viscosities and reasonable molecular weights. This study also clearly shows the relationship between the monomer structure and the properties underlining the major role of the amide content on the copolymer hydrolytic degradation behaviour. Work is in progress to assess the properties and possible applications of these materials.
